# The neutralizing effect of heparin on blood-derived antimicrobial compounds: impact on antibacterial activity and inflammatory response

**DOI:** 10.3389/fimmu.2024.1373255

**Published:** 2024-03-22

**Authors:** Denisa Cont, Stephan Harm, Claudia Schildböck, Claudia Kolm, Alexander K. T. Kirschner, Andreas H. Farnleitner, Matthias Pilecky, Jennifer Zottl, Jens Hartmann, Viktoria Weber

**Affiliations:** ^1^ Department for Biomedical Research, University for Continuing Education Krems, Krems an der Donau, Austria; ^2^ Department of Physiology, Pharmacology and Microbiology, Division Water Quality and Health, Karl Landsteiner University of Health Sciences, Krems an der Donau, Austria; ^3^ Institute of Chemical, Environmental and Bioscience Engineering, Research Group Microbiology and Molecular Diagnostics, Vienna University of Technology, Vienna, Austria; ^4^ Institute for Hygiene and Applied Immunology, Water Microbiology, Medical University of Vienna, Vienna, Austria; ^5^ Research Lab Aquatic Ecosystem Research and Health, University for Continuing Education Krems, Krems an der Donau, Austria; ^6^ Water Cluster Lunz Biological Station, Lunz, Austria

**Keywords:** antibacterial activity, antimicrobial compounds, endotoxin neutralization, host defense peptides, heparin, LPS, pathogenic bacteria

## Abstract

Acting through a combination of direct and indirect pathogen clearance mechanisms, blood-derived antimicrobial compounds (AMCs) play a pivotal role in innate immunity, safeguarding the host against invading microorganisms. Besides their antimicrobial activity, some AMCs can neutralize endotoxins, preventing their interaction with immune cells and avoiding an excessive inflammatory response. In this study, we aimed to investigate the influence of unfractionated heparin, a polyanionic drug clinically used as anticoagulant, on the endotoxin-neutralizing and antibacterial activity of blood-derived AMCs. Serum samples from healthy donors were pre-incubated with increasing concentrations of heparin for different time periods and tested against pathogenic bacteria (*Acinetobacter baumannii*, *Enterococcus faecium*, *Escherichia coli*, *Klebsiella pneumoniae*, *Pseudomonas aeruginosa*, *Staphylococcus aureus*) and endotoxins from *E. coli*, *K. pneumonia*e, and *P. aeruginosa*. Heparin dose-dependently decreased the activity of blood-derived AMCs. Consequently, pre-incubation with heparin led to increased activity of LPS and higher values of the pro-inflammatory cytokines tumor necrosis factor α (TNF-α) and interleukin 6 (IL-6). Accordingly, higher concentrations of *A. baumannii*, *E. coli*, *K. pneumoniae*, and *P. aeruginosa* were observed as well. These findings underscore the neutralizing effect of unfractionated heparin on blood-derived AMCs *in vitro* and may lead to alternative affinity techniques for isolating and characterizing novel AMCs with the potential for clinical translation.

## 1 Introduction

The innate immune system, constitutes the first line of defense against infections, encompassing a diverse array of synergistic mechanisms designed to rapidly recognize and eradicate invading pathogens. As part of this defense, human whole blood contains a vast repertoire of antimicrobial compounds (AMCs), including antimicrobial peptides (AMPs), complement proteins, collectins, and other immune factors (1–4).

AMPs, also known as host defense peptides, are short cationic amphiphilic molecules with a unique combination of anti-inflammatory, antimicrobial, and immunostimulatory properties (5–7). Among the different antimicrobial activities of AMPs, their most prominent role is to function as antibacterial agents against Gram-positive and Gram-negative bacteria (8). The mechanism of action mainly relies on a rapid and direct interaction with the bacterial cell wall (9). These positively charged peptides engage with the negatively charged lipids present on the bacterial surface, i.e., endotoxins (lipopolysaccharides, LPS) in the outer membrane of Gram-negative bacteria or lipoteichoic acid in case of Gram-positive bacteria. This interaction leads to the accumulation of peptides on the membrane, the formation of pores and channels that compromise its integrity, ultimately resulting in membrane collapse and lytic cell death (10–12). Certain AMPs can also translocate into the bacteria and act on intracellular targets, being able to disrupt protein biosynthesis by inhibiting transcription, translation, and protein assembly (9, 13, 14). Defensins, cathelicidins, and bactericidal/permeability-increasing protein (BPI) are prime examples of AMPs found in serum (15, 16).

In the presence of infectious agents, another set of proteins promptly become activated – the complement system and collectins. Activation of the complement cascade results in opsonization of pathogens through the activated product C3b, enhancing phagocytosis. This cascade leads to the formation of a membrane attack complex that inserts into the microbial membrane, resulting in direct lysis. Additionally, short peptides such as C3a and C4a are produced, exhibiting both antimicrobial properties and anaphylactic activity (3, 17). Collectins, a family of C-type lectins, are also capable of recognizing and binding to various pathogens, resulting in antimicrobial activity through membrane disruption, as well as opsonization (18). Mannose-binding lectin (MBL) is a specific example of a collectin found in serum with antimicrobial activity against Gram-negative and Gram-positive bacteria, and viruses (19, 20).

During bacterial division and cell death, LPS can be released into the bloodstream, leading to an interaction with host pattern recognition receptors, such as toll-like receptors (TLRs), triggering immune cells to produce and release cytokines (21). Under certain circumstances, this can result to a dysregulation of the immune response, ultimately resulting in life-threatening conditions, such as sepsis (22–24). Within AMCs, certain molecules, referred to as endotoxin-neutralizing compounds (ENCs), can mitigate inflammatory responses through direct binding and neutralization of LPS, as well as by downregulating the expression of pro-inflammatory cytokines (25–28).

**Figure 1:**
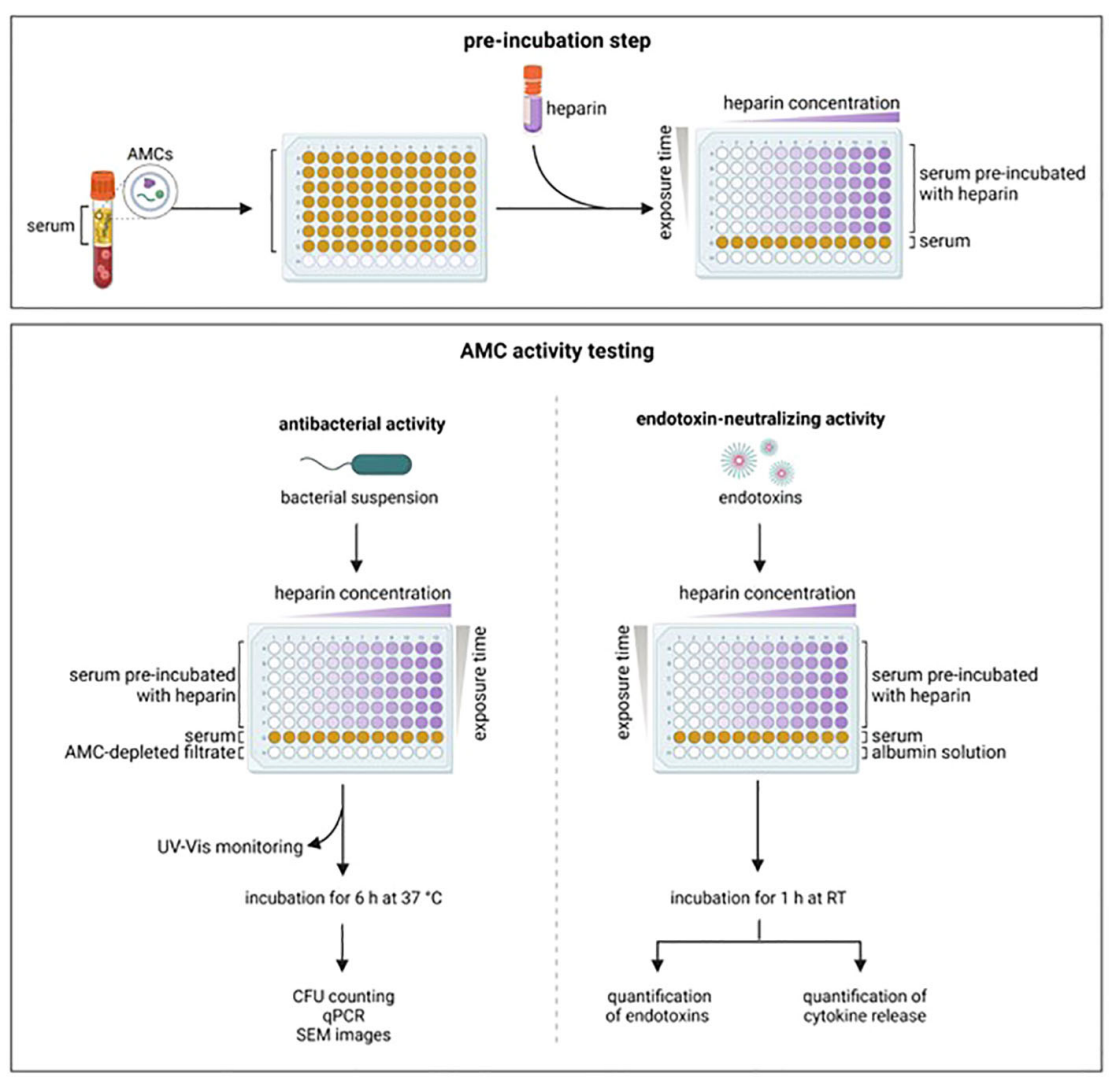
Experimental set-up. Serum from healthy donors was pre-incubated with increasing concentration of heparin and for different time periods. After pre-incubation, the antibacterial and the endotoxin-neutralizing activity were tested. AMCs, antimicrobial compounds; CFU, colony forming units; qPCR, real time polymerase chain reaction; RT, room temperature; SEM, scanning electronic microscope. Created with BioRender.com.

Recent studies have shown that ENCs can be neutralized by unfractionated heparin, therefore restoring the LPS activity in serum/plasma samples (29–31). Heparin, a negatively charged polysaccharide with anticoagulant and anti-inflammatory properties, has clinically used in septic patients (32). We hypothesized that heparin can interfere and neutralize blood-derived AMCs. Therefore, in the present study, we investigated the impact of unfractionated heparin on the antibacterial and endotoxin-neutralizing activity of AMCs from serum samples against pathogenic bacteria (*A. baumannii*, *E. faecium*, *E. coli*, *K. pneumoniae*, *P. aeruginosa*, *S. aureus*) and endotoxins from *E. coli*, *K. pneumonia*e, and *P. aeruginosa* (**Figure 1**).

## 2 Materials and methods

### 2.1 Sample collection, bacterial strains, and lipopolysaccharides

Human whole blood was drawn from healthy volunteer donors into vacutainer tubes (Vacuette CAT Serum Clot Activator tubes, Greiner Bio-One, Kremsmünster, Austria). After clotting, samples were centrifuged at 3500 x g for 10 min, and serum was frozen in aliquots at -20°C until further use. The bacterial strains used were *A. baumannii* (ATCC 19606), *E. faecium* (DSM 20477), *E. coli* (ATCC 25299), *K. pneumoniae* (ATCC 13882), *P. aeruginosa* (NCTC 10662), and *S. aureus* (DSM 20232). These strains were preserved in glycerol stocks at -80°C for long-term storage and reactivated by culture on nutrient agar plates (NA, Carl Roth GmbH & Co. KG, Karlsruhe, Germany) at 37°C overnight, prior to each experiment. LPS from *E. coli* O55:B5, *K. pneumoniae* ATCC 15380, and *P. aeruginosa* ATCC 27316 were obtained from Sigma Aldrich (St. Louis, MO).

### 2.2 AMC-depleted filtrate

Citrated plasma from a pool of 12 healthy donors with same blood group (B-) (Red Cross Blood Center Linz, Austria) was spiked with 500 mM Ca^2+^ and 250 mM Mg^2+^ to induce clotting. Serum was separated from the clot by centrifugation (3500 x g, 10 min) and recirculated through a high-flux filter (Ultraflux EMiC2, Fresenius Medical Care, Bad Homburg, Germany). Filtration conditions and parameters for native serum and the AMC-depleted filtrate are specified in **Supplementary Table 1.**

### 2.3 Endotoxin-neutralizing activity of blood-derived AMCs

Serum samples were independently spiked with 50 ng/mL LPS from *E. coli*, *K. pneumoniae*, and *P. aeruginosa*, respectively, and incubated for 0, 1, 3, and 6 h at room temperature. As control, LPS was spiked into physiological saline solution (Fresenius Medical Care) containing 1% human serum albumin (HSA, Kedrion, Biopharma, Vienna, Austria) and 1 mM Mg^2+^ (Merck, Darmstadt, Germany) (albumin solution). LPS was quantified using the kinetic chromogenic Limulus amebocyte lysate (LAL) assay (Endosafe Endochrome-K, Charles River, Wilmington, MA) according to the instructions of the manufacturer.

### 2.4 Exposure time and concentration of heparin required to inhibit the endotoxin-neutralizing activity of AMCs

To assess the optimal heparin concentration for the maximum inhibition of AMCs, serum samples were pre-incubated with increasing concentrations of heparin (5, 10, 50, 100, and 150 IU/ml unfractionated heparin, Gilvasan Pharma GmbH, Vienna, Austria) for 1 h at room temperature. To assess the influence of exposure time, serum samples were pre-incubated with 100 IU/mL heparin for 0, 1, 3, and 6 h at room temperature. As a control, incubation was performed in albumin solution and in native serum. After pre-incubation, samples were spiked with 50 ng/mL LPS from *E. coli*, *K. pneumoniae*, and *P. aeruginosa*, respectively, and incubated for 1 h at room temperature. LPS was quantified as described above.

### 2.5 Evaluating the established pre-incubation conditions in serum from healthy donors: endotoxin-neutralizing activity

The endotoxin-neutralizing activity was tested directly through quantification of LPS and indirectly through the release of TNF-α and IL-6. Fresh serum samples from six donors were independently pre-incubated with 100 IU/mL heparin for 1 h at room temperature. After incubation, heparin-spiked serum and native serum were incubated for 1 h at room temperature with 50 ng/mL LPS (for LPS quantification) or with 5 ng/mL LPS (for cytokine quantification) from *E. coli*, *K. pneumoniae*, and *P. aeruginosa*. The quantification of LPS was performed using the LAL assay. To assess LPS-induced cytokine release, blood was drawn into vacutainer tubes (Vacuette 9NC Coagulation Trisodium Citrate 3.2% tubes, Greiner bio-one). Plasma was separated from blood cells by centrifugation (3500 x g, 10 min). LPS-spiked samples were mixed at 1:1 ratio with the fresh blood cells and incubated at 37°C. After 4 h, samples were centrifuged (3500 x g, 10 min) and the supernatant was employed to quantify TNF-α and IL-6 by the Luminex Multiplex Bead Array (R&D Systems, Minneapolis, MN), following the protocol provided by the manufacturer, using a Bio-plex-200 Analyzer (Bio-Rad Laboratories, Hercules, CA).

### 2.6 Antibacterial activity of blood-derived AMCs

A bacterial suspension with an optical density of 2.0 ± 0.2 at 600 nm (equivalent to 3x10^8^ colony forming units (CFU)/mL based on McFarland standards) in Luria Broth (LB) medium (Carl Roth) from *A. baumannii*, *E. faecium*, *E. coli*, *K. pneumoniae*, *P. aeruginosa*, and *S. aureus* was prepared and progressively diluted 10-fold with LB until 3x10^3^ CFU/mL. Each bacterial dilution was incubated at 1:1 ratio with serum (overnight at 37°C). Controls were performed in AMC-depleted filtrate. After incubation, bacterial growth was measured indirectly by determining the absorbance at 600 nm.

### 2.7 Exposure time and concentration of heparin required to inhibit the antibacterial activity of AMCs

The influence of heparin on the antibacterial activity was examined using qPCR and kinetic absorbance monitoring. Serum samples were pre-incubated with increasing concentrations of heparin (5, 50, 100, 250 IU/mL) for 0, 4, and 10 h at 37°C. As a control, the same experimental setups were conducted in AMC-depleted filtrate and in native serum. After pre-incubation, samples were mixed with an equal volume of bacterial suspension (3x10^4^ CFU/mL) in Luria Broth (LB, Carl Roth) from *A. baumannii*, *E. coli*, *K. pneumoniae*, and *P. aeruginosa*. Samples were subjected to a kinetic absorbance monitoring for 20 h in a plate reader at 37°C and 600 nm, with measurements taken hourly. The detailed qPCR protocol and the sequence of the in-house designed primers used in this study are given in **Supplementary Table 2.**

### 2.8 Evaluating the established pre-incubation conditions in serum from healthy donors: antibacterial activity

Serum samples from six donors were pre-incubated with 250 IU/mL heparin for 4 h at 37°C. After incubation, heparin-spiked serum and native serum were incubated at a 1:1 ratio with a 3x10^4^ CFU/mL suspension from each strain. The bacterial growth in the samples was monitored using a plate reader for 20 h at 37°C, with measurements recorded hourly at 600 nm. For the qPCR, the CFU counting and scanning electron microscope (SEM) images, after adding the bacterial strains, samples were incubated at 37°C for 6 h and subjected to qPCR and CFU counting. The bacteria integrity following incubation with heparin-spiked serum and native serum was assessed by SEM. Samples were filtered through 0.2 µm membrane filters (Isopore™, Cork, Ireland). The collected bacteria were washed with saline solution and fixed with a 2.5% glutaraldehyde solution (Carl Roth). The fixed samples were gradually dehydrated by increasing ethanol solutions (10-100%) and sputtered with gold (Q150R ES Sputter Coater, Quorum Technologies Ltd., East Sussex, England) prior to the examination with SEM (FlexSEM 1000, Hitachi, Tokyo, Japan).

### 2.9 Statistical analysis

Unless otherwise indicated, all experiments were conducted in duplicates (two donors, two measurements per donor). Statistical tests were carried out using GraphPad Prism 9.3.1 (GraphPad Software, Boston, MA). Normal distribution was checked applying the Kolmogorov-Smirnov Test. Normally distributed data were compared using the t-Test. For non-normally distributed data, the Mann-Whitney Rank Sum Test was used. P-values ≤ 0.05 were considered as statistically significant.

## 3 Results

### 3.1 Endotoxin-neutralizing activity of blood-derived AMCs

**Figure 2:**
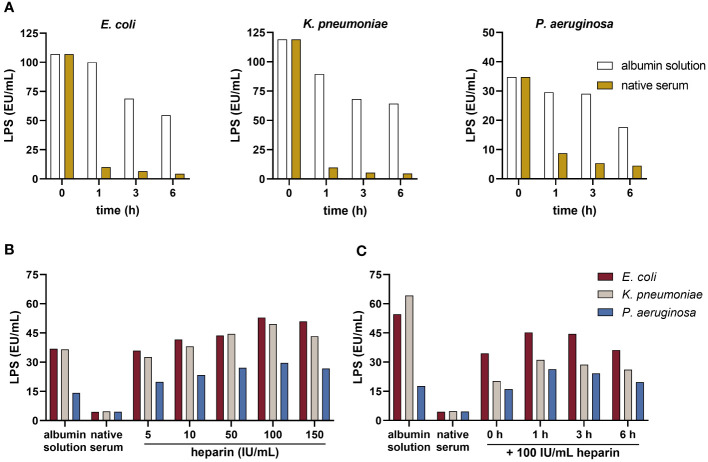
Neutralization of LPS by serum and the influence of heparin on blood-derived AMCs. **(A)** Endotoxin-neutralizing activity: serum and the albumin solution were spiked with 50 ng/mL LPS from *E. coli*, *K. pneumoniae*, and *P. aeruginosa* and incubated for 0, 1, 3, and 6 h at room temperature. LPS was quantified using the kinetic chromogenic LAL assay according to the protocol provided by the manufacturer (n = 2). **(B)** Concentration assessment: serum was pre-incubated with increasing concentrations of heparin (5, 10, 50, 100 and 150 IU/ml) for 1 h at room temperature. The albumin solution and native serum were used as controls. After pre-incubation, samples were spiked with 50 ng/mL LPS from each strain and incubated for 1 h at room temperature. LPS was quantified using the LAL assay (n = 2). **(C)** Exposure time assessment: serum was pre-incubated with 100 IU/mL heparin for 0, 1, 3, and 6 h at room temperature. The albumin solution and native serum were used as controls. After pre-incubation, samples were spiked with 50 ng/mL LPS from each strain and incubated for 1 h at room temperature. LPS was quantified using the LAL assay (n = 2).

In comparison to albumin solution, human serum showed a higher endotoxin-neutralizing capacity, indicated by decreased LPS activity in serum samples (**Figure 2A**) spiked with LPS from *E. coli*, *K. pneumoniae*, or *P. aeruginosa*, respectively. After 1 h, the LPS values of *E. coli*, *K. pneumoniae*, and *P. aeruginosa* were reduced by a factor 9.9, 9.2, and 3.4, respectively, when compared to albumin solution. For all further experiments, a 1-hour incubation of LPS with serum was used.

### 3.2 Exposure time and concentration of heparin required to inhibit the endotoxin-neutralizing activity of AMCs

The results demonstrated a dose-dependent increase in LPS values with rising heparin concentration, reaching maximum LPS values at 100 IU/mL heparin (**Figure 2B**). This was consistent across LPS from all strains tested. Pre-incubating serum with 100 IU/mL heparin led to an increase of the LPS levels by a factor of approximately 11.9, 10.4, and 6.6 for spikes from *E. coli*, *K. pneumoniae*, and *P. aeruginosa*, respectively, compared to native serum. Lower concentrations of heparin reduced endotoxin-neutralizing activity of AMCs as well. Five IU/mL heparin increased the LPS values by a factor of 8.1, 6.8, and 4.4, respectively. Regarding the optimal exposure time, heparin-spiked samples pre-incubated for 1 h showed an increase in the LPS values by a factor of 10.2, 6.5, and 5.8 from *E. coli*, *K. pneumoniae*, and *P. aeruginosa*, respectively, compared to the native serum (**Figure 2C**). LPS values in heparin-spiked serum samples were nearly equivalent to the albumin solution used as control. Based on these findings, for further experiments, 1 h pre-incubation with 100 IU/mL heparin was established.

### 3.3 Evaluating the established pre-incubation conditions in serum from healthy donors: endotoxin-neutralizing activity

**Figure 3:**
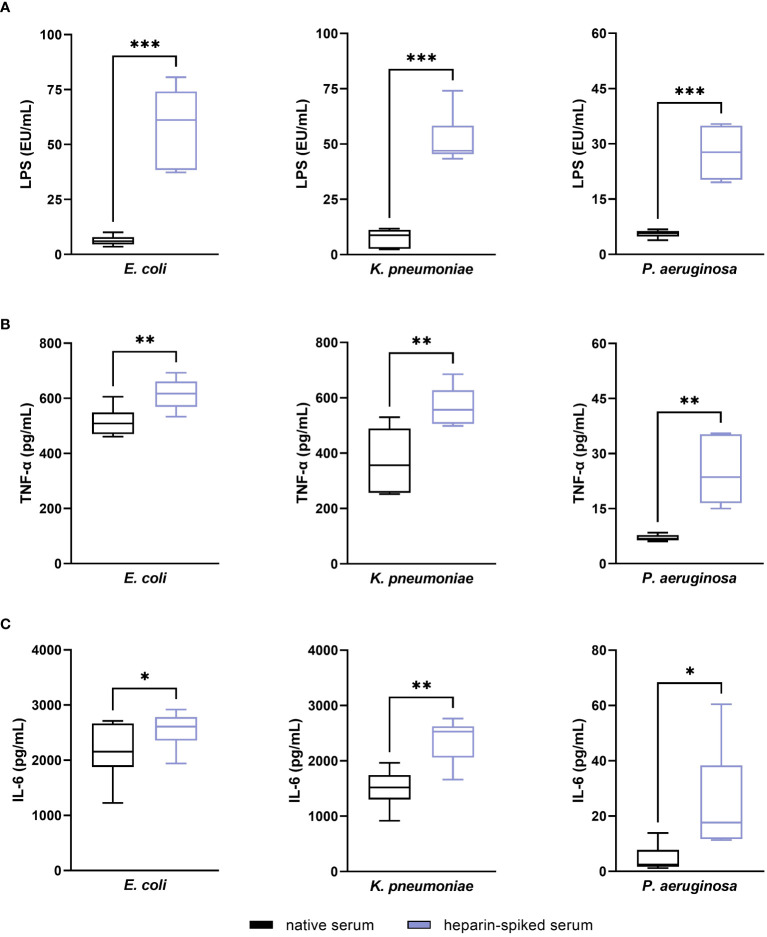
Evaluating the established pre-incubation conditions in serum from healthy donors: endotoxin-neutralizing activity. Serum samples from six different donors were pre-incubated with 100 IU/mL heparin for 1 h at room temperature. After incubation, 50 ng/mL LPS (for LPS quantification) or 5 ng/mL LPS (for cytokine quantification) from *E. coli*, *K. pneumoniae*, and *P. aeruginosa* were added to the heparin-spiked serum and native serum samples and incubated for 1 additional hour at room temperature. LPS was quantified using the LAL test **(A)**. TNF-α **(B)** and IL-6 **(C)** released in response to LPS were quantified as described in the materials and methods section (n = 6). * p ≤ 0.05; ** p ≤ 0.01; *** p ≤ 0.001.

We found a statistically significant difference (p ≤ 0.001) between native and heparin-spiked serum in the recovery of LPS values from *E. coli*, *K. pneumoniae* and *P. aeruginosa* (**Figure 3A**). The pre-incubation with heparin yielded a 9.3-fold increase in LPS values from *E. coli* compared to native serum. In case of *K. pneumoniae* and *P. aeruginosa* LPS, heparin-spiked samples had 6.9 and 4.9-fold increase LPS levels compared to native serum samples. The results from the cytokine quantification showed a significant increase in TNF-α and IL-6 in serum samples incubated with heparin compared to native serum (**Figures 3B, C**).

### 3.4 Antibacterial activity of blood-derived AMCs

Within our experimental conditions, the capacity of serum to inhibit bacterial growth varied depending on the strain. Serum could inhibit the growth of *A. baumannii* and *K. pneumoniae* up to 10^7^ CFU/mL, and for *E. coli* and *P. aeruginosa* up to 10^5^ CFU/mL. Serum did not exhibit significant inhibitory effects on the Gram-positive bacteria tested. Based on this outcome, 10^4^ CFU/mL bacterial suspensions were used for further experiments.

### 3.5 Exposure time and concentration of heparin required to inhibit the antibacterial activity of AMCs

**Figure 4:**
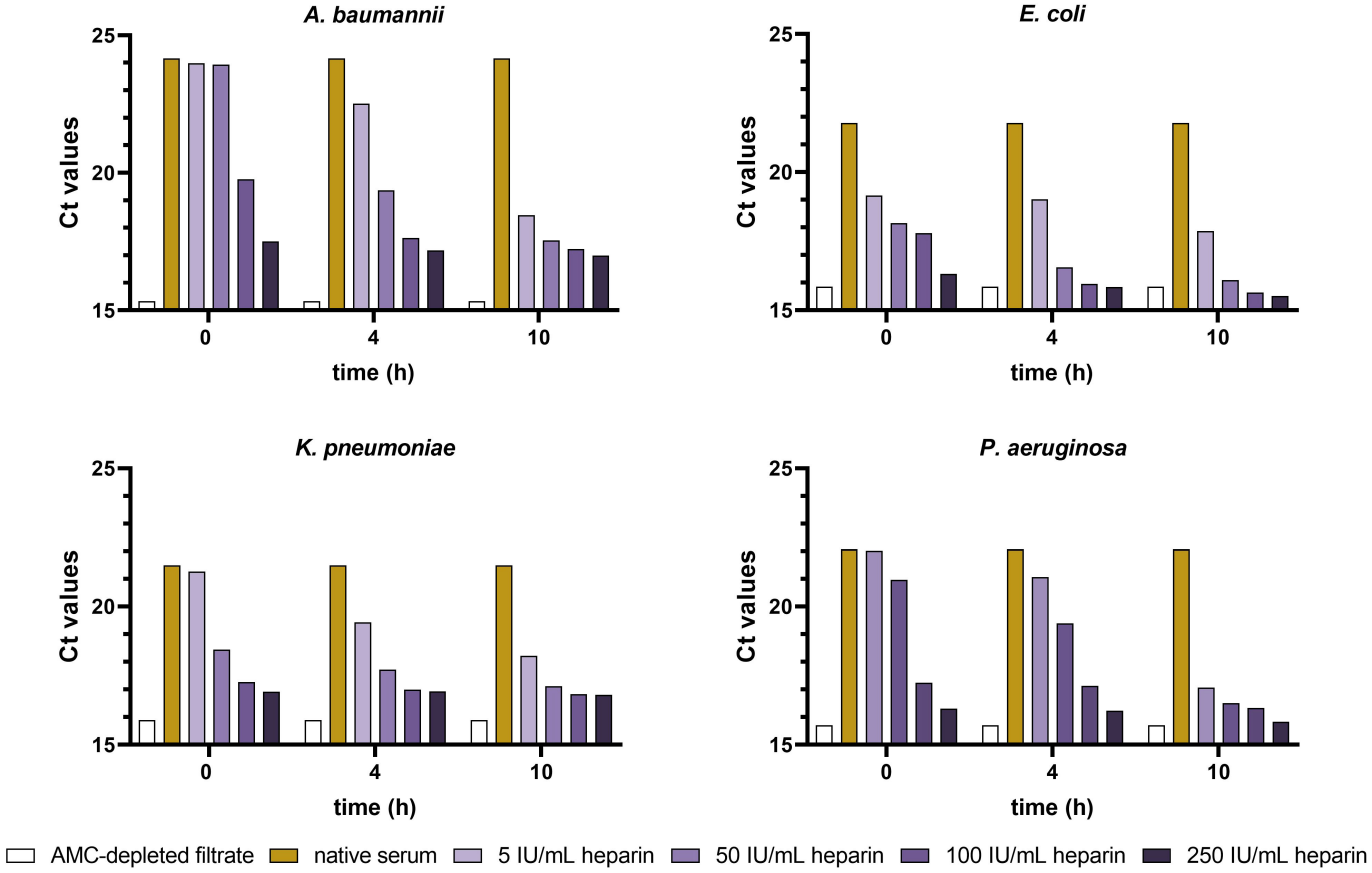
Ct values from *A. baumannii*, *E. coli*, *K. pneumoniae*, and *P. aeruginosa* incubated in heparin-spiked serum and native serum samples. Serum samples were pre-incubated with increasing concentrations of heparin (5, 50, 100, and 250 IU/mL) for 0, 4, and 10 h at 37°C. AMC-depleted filtrate and native serum were used as controls. After pre-incubation, samples were incubated for 6 h with 3x10^4^ CFU/ml suspension of *A. baumannii*, *E. coli*, *K. pneumoniae*, and *P. aeruginosa* at 37°C. Ct values for each strain were determined as described in the materials and methods section (n = 2).

The results from bacterial target quantification (based on qPCR) showed a correlation between the increasing heparin concentrations/pre-incubation times with the growth of *A. baumannii*, *E. coli*, *K. pneumoniae*, and *P. aeruginosa*, compared to native serum (**Figure 4**). Ct values decreased in the Gram-negative strains tested when increasing heparin concentration and pre-incubation times. Concentrations of 100 and 250 IU/mL heparin had an immediate AMC neutralizing effect, with Ct values of 17.2, 15.9, 16.9, and 16.2 (250 IU/ml heparin) in *A. baumannii*, *E. coli*, *K. pneumoniae*, and *P. aeruginosa*, compared to the 24.2, 21.8, 21.5, and 22.07 Ct values of native serum, respectively. In case of 5 and 50 IU/mL, the lowest Ct values were obtained after 10 h incubation. The bacterial growth curves obtained from the kinetic absorbance monitoring in *A. baumannii*, *E. coli*, *K. pneumoniae* and *P. aeruginosa* had the same tendency as the qPCR data (**Supplementary Figure 1**).

### 3.6 Evaluating the established pre-incubation conditions in serum from healthy donors: antibacterial activity

**Figure 5:**
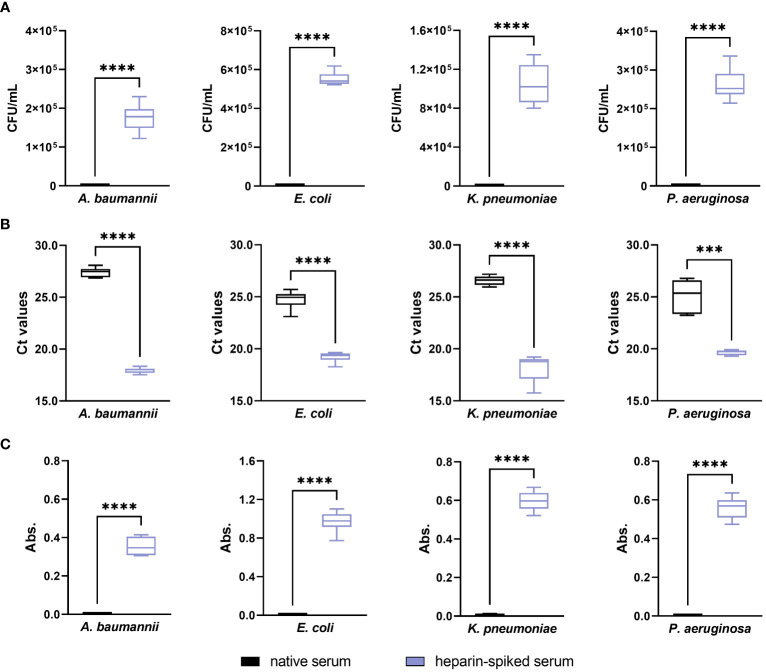
Evaluating the optimized pre-incubation conditions in serum from healthy donors: antibacterial activity. Serum samples from six different donors were pre-incubated with 250 IU/mL heparin for 4 h at 37°C. After incubation, 3x10^4^ CFU/mL suspension of *A. baumannii*, *E. coli*, *K. pneumoniae*, and *P. aeruginosa* were added to the heparin-spiked serum and native serum. Differences between the two groups were analyzed by colony forming units (CFU) counting **(A)**, qPCR **(B)**, and absorbance measurements at 600 nm **(C)** as described in the materials and methods section (n = 6). *** p ≤ 0.001; **** p ≤ 0.0001.

The native serum had a strong antibacterial effect against *A. baumannii*, *E. coli*, *K. pneumoniae*, and *P. aeruginosa* with 0 CFU/mL after 6 h incubation (**Figure 5A**), which was significantly reversed upon pre-incubation with heparin (p ≤ 0.0001). In case of *E. faecium* and *S. aureus*, as already detailed above, native serum did not exhibit an antibacterial effect against the Gram-positive bacteria included in this study. Regarding the qPCR results, the Ct values obtained from the heparin-spiked serum were lower than for native serum, correlating to a higher concentration of bacterial DNA-targets in the samples (**Figure 5B**). Thus, the results obtained with the absorbance measurements (**Figure 5C**) showed the same tendency as the CFU count and the Ct values. In the native serum, an increase in absorbance values was not noticed in the Gram-negative strains, which remained undetectable over the whole period of incubation. In the heparin-spiked samples, however, the measurements showed a progressive increase in the absorbance values.

### 3.7 Visualization of bacterial integrity using SEM

**Figure 6:**
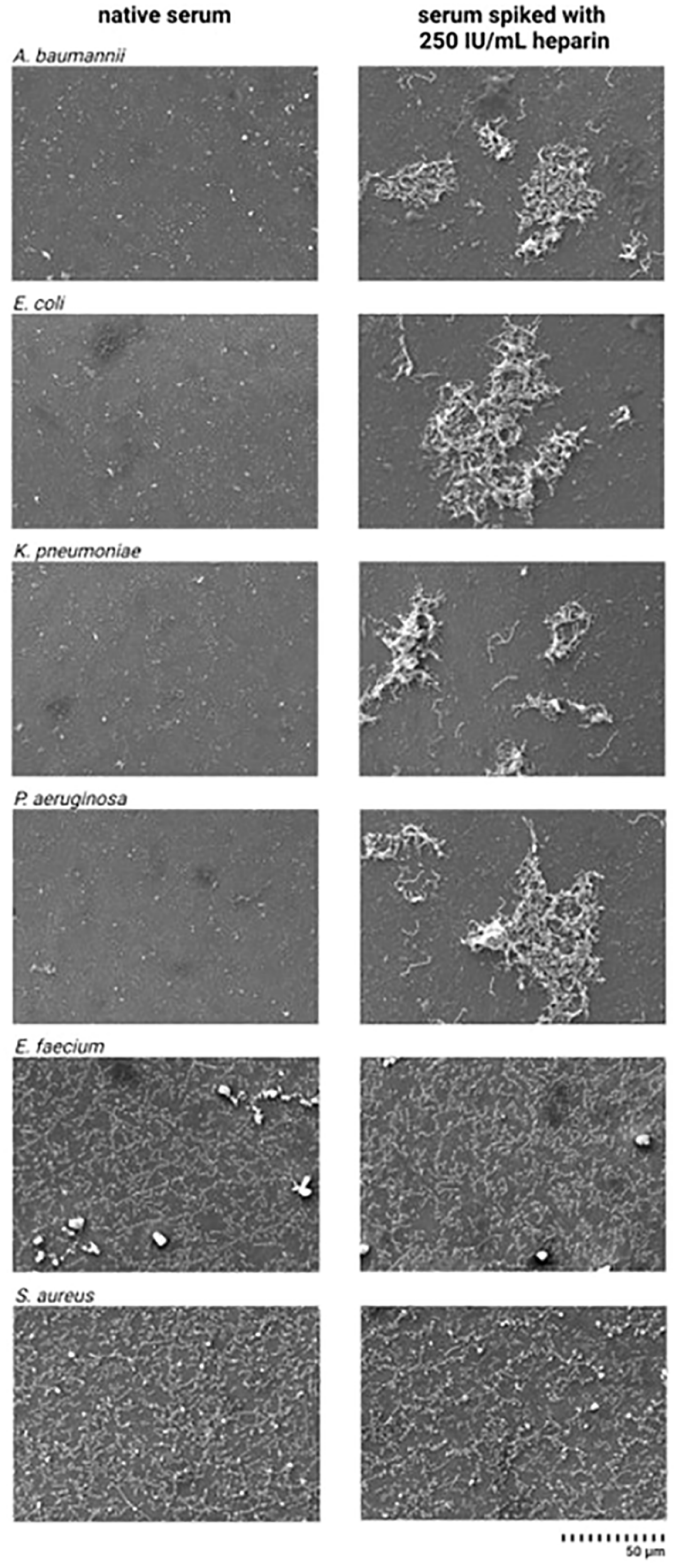
Scanning electron microscope images from pathogenic bacteria incubated in native serum and heparin-spiked serum. Serum samples were pre-incubated with 250 IU/mL heparin for 4 h at 37°C. After incubation, 3x10^4^ CFU/mL suspension of *A. baumannii*, *E. faecium*, *E. coli*, *K. pneumoniae*, *P. aeruginosa*, and *S. aureus* were added to the heparin-spiked serum and native serum. SEM images were prepared as described in the materials and methods section.

Differences in the bacterial integrity of Gram-negative bacteria were observed in the SEM images when comparing the serum samples incubated with heparin and the native serum (**Figure 6**). In the images obtained from the heparin-spiked samples, *A. baumannii*, *E. coli*, *K. pneumoniae*, and *P. aeruginosa* appear to be intact, while in the native serum no morphologically intact bacteria where found, in contrast to *E. faecium* and *S. aureus*.

## 4 Discussion

Human whole blood contains a repertoire of ENCs, AMPs being a crucial constituent within this group (12, 33, 34). These molecules represent a challenge in detecting endotoxins in clinical samples using conventional endotoxin-detection assays (35–37). Our previous research revealed the potential of heparin, a polyanionic anticoagulant, to enable the detection and quantification of endotoxins in blood-derived samples using the Limulus amebocyte lysate assay while also notably enhancing their immunostimulatory properties. Our underlying hypothesis postulated that polyanionic heparin binds to the cationic ENCs, preventing their interaction with endotoxins (negatively charged) (29, 31).

In this study, we assessed the influence of unfractionated heparin on the endotoxin-neutralizing and antibacterial activity of blood-derived AMCs. The antibacterial activity was evaluated against 5 out of 6 ESKAPE pathogens and *E. coli*. The term ESKAPE is an acronym comprising the scientific names of six bacterial pathogens including *E. faecium*, *S. aureus*, *K. pneumoniae*, *A. baumannii*, *P. aeruginosa*, and *Enterobacter* sp. with high clinical relevance (38). Although not officially recognized as part of the ESKAPE pathogens, *E. coli* was also included in the study since is a major cause of bloodstream and urinary tract infections (39). We were able to confirm our hypothesis that unfractionated heparin interferes with and neutralizes blood-derived AMCs, resulting in higher bacterial growth and endotoxin activity in serum samples spiked with heparin, compared to native serum.

We initially evaluated the activity of native serum against the above-mentioned strains and LPS types. Exposing serum to LPS from *E. coli*, *K. pneumoniae*, and *P. aeruginosa* led to a reduction of over 70% in the LPS levels within just one hour. This highlights the rapid and effective capacity of serum to neutralize endotoxins. Regarding the antibacterial activity, serum effectively inhibited the growth of *A. baumannii* and *K. pneumoniae* up to 10^7^ CFU/mL, and for *E. coli* and *P. aeruginosa* up to 10^5^ CFU/mL. However, this effect was not observed for the Gram-positive bacteria tested, i.e., *E. faecium* and *S. aureus.* The pathogenicity of the bacteria found in septicemia depends in part on their ability to evade the bactericidal effect of serum. It is worth noting that within the same bacterial species, serum-sensitive and serum-resistant isolates can be found (40–43). Although certain blood-derived AMPs, such as defensins and cathelicidins, are recognized for their broad-spectrum antibacterial activity against Gram-negative and Gram-positive bacteria (15, 44), this activity within serum was insufficient in the *E. faecium* and *S. aureus* strains we tested. Moreover, it is plausible that AMPs targeting Gram-positive bacteria are stored within the granules of leukocytes and platelets, potentially being released upon the activation of blood cells by Gram-positive associated PAMPs, like lipoteichoic acids or peptidoglycans.

Incubating serum with heparin resulted in a decrease in both endotoxin-neutralizing and antibacterial activities. The highest decrease in the endotoxin neutralization occurred with the exposure of serum to 100 IU/mL heparin for one hour, when compared to native serum. Notably, this effect was evident even at lower heparin concentrations, beginning with 5 IU/mL (lowest heparin concentration tested), being consistent for all three types of LPS tested. In the serum-sensitive pathogenic strains, there was a correlation between the decline of the antibacterial activity of serum and increasing heparin concentration and exposure time. While heparin concentrations of 5 IU/mL required pre-incubation for more than 4 hours for achieving a reduction in the antibacterial activity, higher concentrations required shorter exposure. However, the minimal required concentrations and exposure times depended on the bacterial species. This suggests that each species possesses a different level of sensitivity to AMCs, requiring specific concentrations of heparin to inhibit their antibacterial activity. It is known that serum contains other heparin-binding molecules, such as different proteases/esterase inhibitors, growth factors, chemokines. These molecules may potentially compete with the blood-derived AMCs, whereby only a portion of the available heparin gets bound by AMCs (45). This implies that isolated AMCs may experience the similar neutralization effect with a reduced concentration of heparin required.

To further assess the impact of heparin on the activities of AMCs, we used sera obtained from six healthy donors. Serum pre-incubated with 100 IU/mL heparin for one hour exhibited increased LPS values in the LAL assay compared to the native serum. Additionally, the levels of TNF-α and IL-6 were higher in the heparin-treated serum. Similar effects were noted in the antibacterial activity when serum was pre-incubated with 250 IU/mL heparin for 4 h. In this case, qPCR, colony counts, and absorbance monitoring showed an increased in the concentration of the serum-sensitive strains in the heparin-treated samples compared to native serum. In conclusion, incubation of serum with heparin enables bacterial growth and restores endotoxin activity in comparison to un-spiked serum.

Our data demonstrate that unfractionated heparin effectively neutralizes blood-derived AMCs *in vitro*. The recognition of the affinity of AMCs to heparin and LPS offers the possibility to use affinity-based techniques to isolate AMCs from human whole blood samples. Employing this isolation strategy could facilitate the identification of potential novel AMCs, which may serve as support for novel antibiotics design.

If heparin exhibits a strong affinity for blood-derived AMCs, it is plausible to consider that heparan sulfate, a major component of the endothelial glycocalyx, could yield similar effects. This suggests the presence of an equilibrium between AMCs bound to the blood vessel surface and those free in the serum. Such a mechanism may represent a novel, yet unknown strategy of the innate immune system to establish a protective barrier along the blood vessel surface, preventing the entry of pathogens into the bloodstream and mitigating systemic inflammation during localized infections.

Our findings raised intriguing questions about whether this neutralization effect translates to *in vivo* situations, specifically in patients diagnosed with septicemia who receive heparin as part of the supportive treatment to mitigate the activation of coagulation. It remains to be elucidated whether this effect is specific to unfractionated heparin or if other anticoagulants, such as low molecular weight heparin, could yield to similar outcomes. Further research in this area could not only have the potential to refine the use of anticoagulants in septicemia but also offers insights into novel therapeutic strategies that could benefit patients facing similar clinical conditions.

## Data availability statement

The raw data supporting the conclusions of this article will be made available by the authors, without undue reservation.

## Ethics statement

The studies involving humans were approved by Ethics Committee of the University for Continuing Education Krems (EK GZ 13/2015-2018). The studies were conducted in accordance with the local legislation and institutional requirements. The participants provided their written informed consent to participate in this study.

## Author contributions

DC: Conceptualization, Formal analysis, Investigation, Methodology, Visualization, Writing – original draft, Writing – review & editing, Validation. SH: Conceptualization, Investigation, Methodology, Project administration, Validation, Writing – review & editing, Formal analysis. CS: Conceptualization, Investigation, Methodology, Resources, Validation, Writing – review & editing. CK: Conceptualization, Methodology, Writing – review & editing, Validation. AK: Conceptualization, Methodology, Writing – review & editing, Validation. AF: Conceptualization, Funding acquisition, Supervision, Writing – review & editing. MP: Investigation, Methodology, Writing – review & editing, Conceptualization. JZ: Investigation, Methodology, Writing – review & editing, Conceptualization. JH: Conceptualization, Funding acquisition, Project administration, Supervision, Writing – review & editing. VW: Funding acquisition, Project administration, Supervision, Writing – review & editing, Conceptualization.

## References

[1] Boman HG. Gene-encoded peptide antibiotics and the concept of innate immunity: an update review. Scand J Immunol. (1998) 48:15–25. doi: 10.1046/j.1365-3083.1998.00343.x 9714406

[2] Lehrer RI Ganz T. Antimicrobial peptides in mammalian and insect host defence. Curr Opin Immunol. (1999) 11:23–7. doi: 10.1016/s0952-7915(99)80005-3 10047545

[3] Nordahl EA Rydengård V Nyberg P Nitsche DP Mörgelin M Malmsten M. Activation of the complement system generates antibacterial peptides. Proc Natl Acad Sci USA. (2004) 101:16879–84. doi: 10.1073/pnas.0406678101 PMC534732 15550543

[4] Fearon DT Locksley RM. The instructive role of innate immunity in the acquired immune response. Science. (1996) 272:50–3. doi: 10.1126/science.272.5258.50 8600536

[5] Jenssen H Hamill P Hancock RE. Peptide antimicrobial agents. Clin Microbiol Rev. (2006) 19:491–511. doi: 10.1128/CMR.00056-05 16847082 PMC1539102

[6] Hancock RE Sahl HG. Antimicrobial and host-defense peptides as new anti-infective therapeutic strategies. Nat Biotechnol. (2006) 24:1551–7. doi: 10.1038/nbt1267 17160061

[7] Hamill P Brown K Jenssen H Hancock RE. Novel anti-infectives: is host defence the answer? Curr Opin Biotechnol. (2008) 19:628–36. doi: 10.1016/j.copbio.2008.10.006 19000763

[8] Hancock RE Diamond G. The role of cationic antimicrobial peptides in innate host defences. Trends Microbiol. (2000) 8:402–10. doi: 10.1016/s0966-842x(00)01823-0 10989307

[9] Mahlapuu M Håkansson J Ringstad L Björn C. Antimicrobial peptides: an emerging category of therapeutic agents. Front Cell Infect Microbiol. (2016) 6:194. doi: 10.3389/fcimb.2016.00194 28083516 PMC5186781

[10] Mookherjee N Anderson MA Haagsman HP Davidson DJ. Antimicrobial host defence peptides: functions and clinical potential. Antimicrobial Host defence peptides: functions Clin potential. (2020) 19:311–32. doi: 10.1038/s41573-019-0058-8 32107480

[11] Sochacki KA Barns KJ Bucki R Weisshaar JC. Real-time attack on single *Escherichia coli* cells by the human antimicrobial peptide LL-37. Proc Natl Acad Sci U.S.A. (2011) 108:E77–81. doi: 10.1073/pnas.1101130108 PMC3080975 21464330

[12] Huan Y Kong Q Mou H Yi H. Antimicrobial peptides: classification, design, application and research progress in multiple fields. Front Microbiol. (2020) 11:582779. doi: 10.3389/fmicb.2020.582779 33178164 PMC7596191

[13] Kobayashi S Chikushi A Tougu S Imura Y Nishida M Yano Y. Membrane translocation mechanism of the antimicrobial peptide buforin 2. Biochemistry. (2004) 43:15610–6. doi: 10.1021/bi048206q 15581374

[14] Mardirossian M Grzela R Giglione C Meinnel T Gennaro R Mergaert P. The host antimicrobial peptide Bac71-35 binds to bacterial ribosomal proteins and inhibits protein synthesis. Chem Biol. (2014) 21:1639–47. doi: 10.1016/j.chembiol.2014.10.009 25455857

[15] Levy O. Antimicrobial proteins and peptides of blood: templates for novel antimicrobial agents. Blood. (2000) 96:2664–72. doi: 10.1182/blood.V96.8.2664.h8002664_2664_2672 11023496

[16] Routsias JG Marinou D Mavrouli M Tsakris A Pitiriga V. Serum β-defensin 2, A novel biomarker for the diagnosis of acute infections. Diagnostics (Basel). (2023) 13:1885. doi: 10.3390/diagnostics13111885 37296737 PMC10252252

[17] Heesterbeek DAC Angelier ML Harrison RA Rooijakkers SHM. Complement and bacterial infections: from molecular mechanisms to therapeutic applications. J Innate Immun. (2018) 10:455–64. doi: 10.1159/000491439 PMC6784045 30149378

[18] Grubor B Meyerholz DK Ackermann MR. Collectins and cationic antimicrobial peptides of the respiratory epithelia. Vet Pathol. (2006) 43:595–612. doi: 10.1354/vp.43-5-595 16966437 PMC2786072

[19] Jack DL Turner MW. Anti-microbial activities of mannose-binding lectin. Biochem Soc Trans. (2003) 31:753–7. doi: 10.1042/bst0310753 12887297

[20] Mayer S Moeller R Monteiro JT Ellrott K Josenhans C Lepenies B. C-type lectin receptor (CLR)-fc fusion proteins as tools to screen for novel CLR/bacteria interactions: an exemplary study on preselected campylobacter jejuni isolates. Front Immunol. (2018) 9:213. doi: 10.3389/fimmu.2018.00213 29487596 PMC5816833

[21] Sun Y Shang D. Inhibitory effects of antimicrobial peptides on lipopolysaccharide-induced inflammation. Mediators Inflammation. (2015) 2015:167572. doi: 10.1155/2015/167572 PMC4647054 26612970

[22] Lyle NH Pena OM Boyd JH Hancock RE. Barriers to the effective treatment of sepsis: antimicrobial agents, sepsis definitions, and host-directed therapies. Ann N Y Acad Sci. (2014) 1323:101–14. doi: 10.1111/nyas.12444 24797961

[23] Cavaillon JM Singer M Skirecki T. Sepsis therapies: learning from 30 years of failure of translational research to propose new leads. EMBO Mol Med. (2020) 12:e10128. doi: 10.15252/emmm.201810128 32176432 PMC7136965

[24] Salomao R Brunialti MK Rapozo MM Baggio-Zappia GL Galanos C Freudenberg M. Bacterial sensing, cell signaling, and modulation of the immune response during sepsis. Shock. (2012) 38:227–42. doi: 10.1097/SHK.0b013e318262c4b0 22777111

[25] Mookherjee N Brown KL Bowdish DM Doria S Falsafi R Hokamp K. Modulation of the TLR-mediated inflammatory response by the endogenous human host defense peptide LL-37. J Immunol. (2006) 176:2455–64. doi: 10.4049/jimmunol.176.4.2455 16456005

[26] Brook M Tomlinson GH Miles K Smith RW Rossi AG Hiemstra PS. Neutrophil-derived alpha defensins control inflammation by inhibiting macrophage mRNA translation. Proc Natl Acad Sci U.S.A. (2016) 113:4350–5. doi: 10.1073/pnas.1601831113 PMC4843457 27044108

[27] Hemshekhar M Anaparti V Mookherjee N. Functions of cationic host defense peptides in immunity. Pharm (Basel). (2016) 9:40. doi: 10.3390/ph9030040 PMC5039493 27384571

[28] Hilchie AL Wuerth K Hancock RE. Immune modulation by multifaceted cationic host defense (antimicrobial) peptides. Nat Chem Biol. (2013) 9:761–8. doi: 10.1038/nchembio.1393 24231617

[29] Harm S Schildböck C Strobl K Hartmann J. An *in vitro* study on factors affecting endotoxin neutralization in human plasma using the Limulus amebocyte lysate test. Sci Rep. (2021) 11:4192. doi: 10.1038/s41598-021-83487-4 33603020 PMC7893160

[30] Harm S Lohner K Fichtinger U Schildböck C Zottl J Hartmann J. Blood compatibility-an important but often forgotten aspect of the characterization of antimicrobial peptides for clinical application. Int J Mol Sci. (2019) 20:5426. doi: 10.3390/ijms20215426 31683553 PMC6862080

[31] Harm S Schildböck C Cont D Weber V. Heparin enables the reliable detection of endotoxin in human serum samples using the Limulus amebocyte lysate assay. Sci Rep. (2024) 14:2410. doi: 10.1038/s41598-024-52735-8 38287051 PMC10825173

[32] Li X Ma X. The role of heparin in sepsis: much more than just an anticoagulant. Br J Haematol. (2017) 179:389–98. doi: 10.1111/bjh.14885 28832958

[33] Berger D Ott S Schmidt UM Bölke E Seidelmann M Beger HG. Determination of endotoxin-neutralizing capacity of plasma in postsurgical patients. Eur Surg Res. (1996) 28:130–9. doi: 10.1159/000129450 8834371

[34] Bennett-Guerrero E Barclay GR Weng PL Bodian CA Feierman DE Vela-Cantos F. Endotoxin-neutralizing capacity of serum from cardiac surgical patients. J Cardiothorac Vasc Anesth. (2001) 15:451–4. doi: 10.1053/jcan.2001.24980 11505348

[35] Elin RJ Robinson RA Levine AS Wolff SM. Lack of clinical usefulness of the limulus test in the diagnosis of endotoxemia. N Engl J Med. (1975) 11:521–4. doi: 10.1056/NEJM197509112931102 1152876

[36] Wong J Davies N Jeraj H Vilar E Viljoen A Farrington K. A comparative study of blood endotoxin detection in haemodialysis patients. J Inflammation (Lond). (2016) 13. doi: 10.1186/s12950-016-0132-5 PMC4967300 27478413

[37] Stumacher RJ Kovnat MJ McCabe WR. Limitations of the usefulness of the Limulus assay for endotoxin. N Engl J Med. (1973) 288:1261–4. doi: 10.1056/NEJM197306142882402 4574106

[38] Rice LB. Federal funding for the study of antimicrobial resistance in nosocomial pathogens: no ESKAPE. J Infect Dis. (2008) 197:1079–81. doi: 10.1086/533452 18419525

[39] Cassini A Högberg LD Plachouras D Quattrocchi A Hoxha A Simonsen GS. Attributable deaths and disability-adjusted life-years caused by infections with antibiotic-resistant bacteria in the EU and the European Economic Area in 2015: a population-level modelling analysis. Lancet Infect Dis. (2019) 19:56–66. doi: 10.1016/S1473-3099(18)30605-4 30409683 PMC6300481

[40] Traub WH. Assay of the antibiotic activity of serum. Appl Microbiol. (1969) 18:51–6. doi: 10.1128/am.18.1.51-56.1969 PMC377884 4309084

[41] Flournoy DJ. Bactericidal effects of human sera versus pathogens. Experientia. (1980) 36:192–3. doi: 10.1007/BF01953725 7371755

[42] Schoolnik GK Buchanan TM Holmes KK. Gonococci causing disseminated gonococcal infection are resistant to the bactericidal action of normal human sera. J Clin Invest. (1976) 58:1163–73. doi: 10.1172/JCI108569 PMC333284 825532

[43] Hastings CJ Syed SS Marques CN. Subversion of the complement system by pseudomonas aeruginosa. J Bacteriol. (2023) 205:e0001823. doi: 10.1128/jb.00018-23 37436150 PMC10464199

[44] Lehrer RI Ganz T. Defensins: endogenous antibiotic peptides from human leukocytes. Ciba Found Symp. (1992) 171:276–93. doi: 10.1002/9780470514344.ch16 1302183

[45] Muñoz EM Linhardt RJ. Heparin-binding domains in vascular biology. Arterioscler Thromb Vasc Biol. (2004) 24:1549–57. doi: 10.1161/01.ATV.0000137189.22999.3f PMC4114236 15231514

