# Supplementary material for: The neutralizing effect of heparin on blood-derived antimicrobial compounds: impact on antibacterial activity and inflammatory response
Source: Front Immunol. 2024 Mar 22;15:1373255. doi: 10.3389/fimmu.2024.1373255 (PMC10995223; doi:10.3389/fimmu.2024.1373255)
Supplement: Supplementary file 1 [file DataSheet_1.docx]

Supplementary Material

The neutralizing effect of heparin on blood-derived antimicrobial compounds: impact on antibacterial activity and inflammatory response

**Denisa Cont, Stephan Harm*, Claudia Schildböck, Claudia Kolm, Alexander K. T. Kirschner, Andreas H. Farnleitner, Matthias Pilecky, Jennifer Zottl, Jens Hartmann, Viktoria Weber**

*** Correspondence:** [stephan.harm@donau-uni.ac.at](mailto:stephan.harm@donau-uni.ac.at)

**Supplementary Table 1. Filtration conditions and parameters for native serum and the AMC-depleted filtrate.** Citrated plasma from a pool of 12 healthy donors with same blood group (B-) (Red Cross Blood Center Linz, Austria) was spiked with 500 mM Ca^2+^ and 250 mM Mg^2+^ to induce clotting. Serum was separated from the clot by centrifugation (3500 x g, 10 min) and recirculated through a high-flux filter (Ultraflux EMiC2, Fresenius Medical Care, Bad Homburg, Germany) with a serum flow rate of 300 mL/min and a filtrate flow rate of 30 mL/min. Ultraflux EMiC2 filter is a high-flux filter for continuous hemodialysis with sieving coefficients for myoglobin (18 kDa) and albumin (66 kDa) of 80 and 0.1%, respectively. Albumin, LDL, myoglobin, and total protein were monitored by Cobas c311 analyzer (Roche Diagnostics, Rotkreuz, Switzerland) using Albumin Gen.2 (ref. 03183688122), LDL-Cholesterol plus 2nd generation (ref. 03038866322), Tina-quant Myoglobin Gen.2 (ref. 04580010190), and Total Protein Gen.2 (ref. 03183734190) Cobas c311 kits, respectively. Calcium and magnesium were quantified using an electrolyte analyzer (NOVA8 CRT, NOVA Biomedical, Waltham, MA). The endotoxin-neutralizing activity was tested against lipopolysaccharide (LPS) from *E. coli* O55:B5. Native serum and the AMC-depleted filtrate were spiked with 50 ng/mL LPS and left incubating for 1 h at room temperature. After incubation, LPS was quantified using the Limulus amebocyte lysate (LAL) assay (Endosafe Endochrome-K, Charles River, Wilmington, MA) following the protocol provided by the manufacturer.

|  | | **Native serum** | **AMC-depleted filtrate** |
| --- | --- | --- | --- |
| **Albumin** | g/dL | 3.76 | 0.31 |
| **Ca^2+^** | mmol/L | 1.26 | 1.15 |
| **LPS** | EU/mL | 7.54 | 94.53 |
| **LDL** | mg/mL | 78.2 | 0.30 |
| **Mg^2+^** | mmol/L | 0.67 | 0.55 |
| **Myoglobin** | U/L | 23.10 | 15.00 |
| **Total protein** | g/mL | 6.10 | 0.60 |

**Supplementary Table 2.** **The** **qPCR protocol and sequence of the in-house design primers used for the bacterial DNA amplification.** For the quantification of the bacterial DNA, the NucleoSpin 8 Virus Kit (Macherey-Nagel, Düren, Germany) was used for the DNA extraction and purification. Samples were centrifuged at 4600 x g for 10 min. Once the supernatant was removed, equal volumes of RNAse-free water and 0.1 mm zirconium beads (Carl Roth, Karlsruhe, Germany) were added to the bacterial pellet. Then, the Lysis Buffer RAV1 was added to each sample in 1:1 ratio and placed on an ultrasonic bath for 10 min. After the ultrasonication, iced ethanol was added (1:1 ratio). The remaining extraction procedure was conduct as specified by the manufacturer. For the qPCR, 15 µL of a PCR mix was added to 5 µL samples. PCR mix was prepared with 150 µL PowerTrack^TM^ SYBR^TM^ Green Master Mix (Applied biosystems, Waltham, MA), 75 µL RNAse-free water, 5 µL forward primer (200 nM), and 5 µL reverse primer (200 nM). The primers used in *Acinetobacter baumannii*, *Escherichia coli*, *Enterococcus faecium*, *Klebsiella pneumoniae*, and *Pseudomonas aeruginosa* specifically amplifies the 16S rRNA gene. The primers used in *Staphylococcus aureus* specifically amplifies collagenase gene. The sensibility and specificity of the in-house genes were previously tested and validated. The qPCR was running on a Light Cycler 96 thermocycler (Roche, Basel, Switzerland) for 45 cycles with the following amplifications conditions: denaturation at 95 °C for 10 sec, annealing at 54 °C for 60 sec, and extension at 72 °C for 15 sec. RNA-free water and bacterial suspension in AMC-depleted filtrate were used as controls. All amplification reactions were run in duplicates, including no template controls to check for contamination.

| **Target species** | **Forward Primer (5’ – 3’)** | **Reverse Primer (5’ – 3’)** | **Amplicon size (bp)** |
| --- | --- | --- | --- |
| ***A. baumannii*** | GCG AGG AGG AGG CTA CTT TAG TTA | TCC TCT CCC ATA CTC TAG CTC AC | 228 |
| ***E. coli*** | GAG GAA GGG AGT AAA GTT AAT ACC TTT | CCC CCT CTA CGA GAC TCA AGC T | 224 |
| ***E. faecium*** | ATA CAT GCA AGT CGA ACG CTT CTT T | TCC ATC AGC GAC ACC CGA AAG | 197 |
| ***K. pneumoniae*** | AGC GGG GAG GAA GGC GAT G | TCT CCT TTG AGT TCC CGG CCT A | 717 |
| ***P. aeruginosa*** | GTT AAT ACC TTG CTG TTT TGA CGT TAC CA | CCT CTA CCG TAC TCT AGC TCA GT | 206 |
| ***S. aureus*** | AAA ACT CTG TTA TTA GGG AAG AAC ATA T | TAC CAG GGT ATC TAA TCC TGT TTG | 373 |

**Supplementary Figure 1. Exposure time and concentration of heparin required to inhibit the antibacterial activity of AMCs**. Serum samples were pre-incubated with increasing concentrations of heparin (5, 50, 100, and 250 IU/mL) for 0, 4, and 10 h at 37 °C. AMC-depleted filtrate and native serum were used as controls. After pre-incubation, samples were incubated for 6 h with 3x10^4^ CFU/ml suspension of *A. baumannii* (**A**), *E. coli* (**B**), *K. pneumoniae* (**C**), and *P. aeruginosa* (**D**). Samples were subjected to a kinetic absorbance monitoring for 24 h at 600 nm and 37 °C.
